# Adaptation, implementation, and mixed methods evaluation of an interprofessional modular clinical practice guideline for delirium management on an inpatient palliative care unit

**DOI:** 10.1186/s12904-022-01010-6

**Published:** 2022-07-16

**Authors:** Shirley H. Bush, Elise Skinner, Peter G. Lawlor, Misha Dhuper, Pamela A. Grassau, José L. Pereira, Alistair R. MacDonald, Henrique A. Parsons, Monisha Kabir

**Affiliations:** 1 Department of Medicine, Division of Palliative Care, University of Ottawa, Ottawa, ON Canada; 2 Bruyère Research Institute, Ottawa, ON Canada; 3 Clinical Epidemiology Program, Ottawa Hospital Research Institute, Ottawa, ON Canada; 4 Department of Palliative Medicine, Bruyère Continuing Care, 43 Bruyère Street, Ottawa, ON K1N 5C8 Canada; 5 Faculty of Medicine, University of Toronto, Toronto, ON Canada; 6 School of Social Work, Carleton University, Ottawa, ON Canada; 7 Division of Palliative Care, Department of Family Medicine, McMaster University, Hamilton, ON Canada; 8 Pallium Canada, Ottawa, ON Canada; 9 Perth and Smiths Falls District Hospital, Smiths Falls, ON Canada; 10 The Ottawa Hospital, Ottawa, ON Canada

**Keywords:** Clinical practice guideline, Implementation, Delirium, Palliative care, Interprofessional, Quality improvement, Knowledge translation, Mixed methods, Educational activities, Learning

## Abstract

**Background:**

Using delirium clinical guidelines may align interprofessional clinical practice and improve the care of delirious patients and their families. The aim of this project was to adapt, implement and evaluate an interprofessional modular delirium clinical practice guideline for an inpatient palliative care unit.

**Methods:**

The setting was a 31-bed adult inpatient palliative care unit within a university-affiliated teaching hospital. Participants for the evaluation were interprofessional team members. Using integration of guideline adaptation and an education initiative, an interprofessional guideline adaptation group developed a face-to-face ‘starter kit’ module and four online self-learning modules. The mixed methods evaluation comprised pre-and post-implementation review of electronic patient records, an online survey, and analysis of focus groups/ interviews using an iterative, inductive thematic analysis approach.

**Results:**

Guideline implementation took 12 months. All palliative care unit staff attended a ‘starter kit’ session. Overall completion rate of the four e-Learning modules was 80.4%. After guideline implementation, nursing documentation of non-pharmacological interventions occurring before medication administration was observed. There was 60% less scheduled antipsychotic use and an increase in ‘as needed’ midazolam use. The online survey response rate was 32% (25/77). Most participants viewed the guideline’s implementation favourably. Six key themes emerged from the qualitative analysis of interviews and focus groups with ten participants: prior delirium knowledge or experiences, challenges of facilitating change, impacts on practice, collaborative effort of change, importance of standardized guidelines, and utility of guideline elements.

**Conclusions:**

Guideline implementation warrants concerted effort, time, and management support. Interprofessional team support facilitates the modular approach of guideline adaptation and implementation, leading to a change in clinical practice.

**Supplementary Information:**

The online version contains supplementary material available at 10.1186/s12904-022-01010-6.

## Background

Delirium is a complex neurocognitive syndrome arising from global organic cerebral dysfunction. It is underdiagnosed, distressing for patients and families, and is associated with increased patient morbidity and mortality [1]. Delirium is present in one third of patients at inpatient palliative care unit admission, and increases to 88% in the last hours or days of life [2]. The hallmark features of delirium are a disturbance in attention, awareness, and cognition [3]. Delirium severity fluctuates over a 24-h period and nursing delirious patients can be extremely challenging, especially at night [4].

Clinical practice guidelines (hereafter referred to as ‘guidelines’) have been defined as “systematically developed statements to assist practitioner and patient decisions about appropriate healthcare for specific clinical circumstances” [5]. Guidelines may help standardise clinical practice, but need to be useful, applicable and relevant [6]. However, guidelines alone are insufficient to improve quality of care and the transfer of pharmacological and behaviour change interventions among healthcare providers into routine clinical practice do not always go as planned [7]. Evidence-based guidelines are rarely adopted successfully leading to inconsistent care practices, and possibly undesired health outcomes [8]. Guidelines should be accessible, share up-to-date evidence, use a standardised presentation that is easy to follow and not overly long, in addition to having a practical implementation process [9, 10]. Although several comprehensive delirium guidelines have been developed, few studies have evaluated their implementation [11].

Healthcare providers often report both delirium knowledge gaps and lack of easy access to clinical guidelines and system processes [12–15]. An interprofessional team approach towards the multicomponent management of delirium can improve quality of care [16]. Interprofessional delirium education interventions should align with organizational needs [17] and e-Learning is an effective and time-efficient intervention for large groups [18]. Family members of patients with delirium also benefit from educational and psychological support [19–21].

The impetus for this project was prompted by a serious incident and expressed family concerns regarding delirium care on our inpatient palliative care unit. The unit’s management committee identified three quality issues: gaps in identifying delirium, a need to standardise practice in the pharmacological management of delirium, and improved interprofessional team communication regarding delirium with patients, family members, and other team members. Secondly, in a previous local staff survey, respondents expressed a desire for guidelines to be “practice-oriented; brief; and consistently accessible”. Thirdly, newly emerging data from a large multicentre randomised clinical trial of antipsychotics to manage targeted delirium symptoms in palliative care patients demonstrated worsening of these symptoms with antipsychotics compared to placebo [22]. Publication of these trial results together with the findings of a systematic review and meta-analysis, which concluded that the use of antipsychotics was not associated with a change in delirium severity or duration [23], prompted a re-evaluation of the symptomatic management of delirium with antipsychotics in palliative care.

To improve the care of patients with delirium, our project aimed to adapt, implement, and evaluate a delirium guideline for the interprofessional team on our inpatient palliative care unit.

## Methods

### Setting and context

The project setting was a 31-bed, adult inpatient palliative care unit in a university-affiliated teaching hospital in Ottawa, Canada. In addition to physicians, registered nurses and registered practical nurses, the regular members of the interprofessional team are a pharmacist, social worker, and spiritual care provider. Other unit staff include ward clerks and a porter. Rotating medical residents and students (medical and nursing) contribute to patient care, and volunteers also provide unit support.

Ethics approval was obtained from the Bruyère Research Ethics Board (#M16-15–028) on June 16, 2015 and the Ottawa Health Science Network Research Ethics Board (#20150416-01H) on July 6, 2015. Interprofessional team member participants provided written informed consent.

### Framework and reporting

The framework used for this project was the Knowledge to Action (KTA) Framework [24] with integration of guideline adaptation using CAN-IMPLEMENT© Version 3.0 [6, 25]. CAN-IMPLEMENT’s recommendation of “Think Implementation” was used throughout the guideline adaptation process and module development.

This report follows the Standards for Quality Improvement Reporting Excellence (SQUIRE) 2.0 Guidelines [26] and uses items from the Template for Intervention Description and Replication (TIDieR) checklist [27] for the education intervention. The completed SQUIRE 2.0 and TIDieR checklists are provided as Additional files (see Additional files 1 and 2 respectively).

### Process of guideline adaptation

**Fig. 1:**
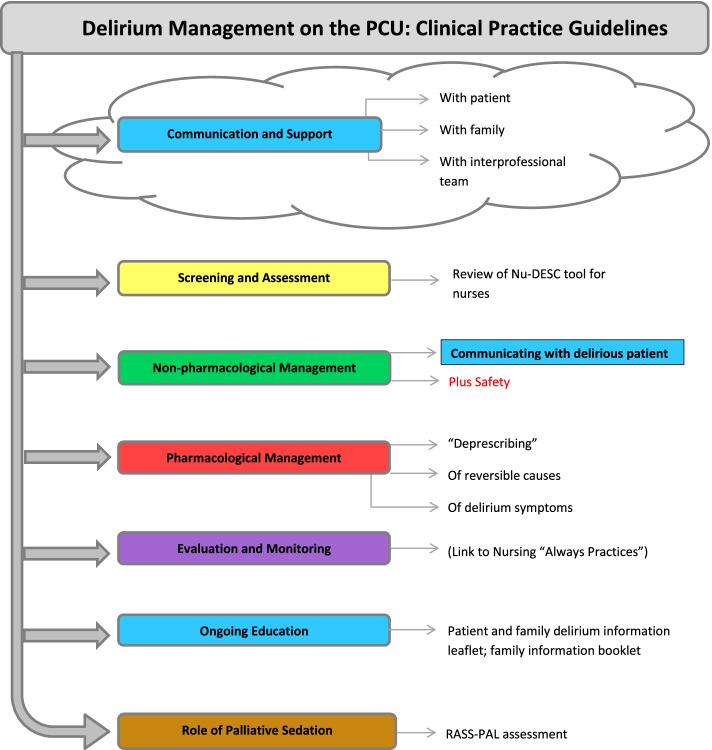
Depiction of the colour-coded modules for the interprofessional delirium guideline. The Silver box called “Delirium management on the PCU: Clinical Practice Guidelines” represents the ‘Starter Kit’ module. The initial Blue box called “Communication and Support” represents a major overarching aim of this project. At the time of this project, formal implementation of the RASS-PAL on the PCU was planned as part of a subsequent e-Learning module on palliative sedation, with content adapted from a regional palliative sedation guideline. Abbreviations: Nu-DESC: Nursing Delirium Screening Scale [32]; PCU: palliative care unit; RASS-PAL: Richmond Agitation-Sedation Scale, palliative version [33]

A systematic appraisal of delirium guidelines was first conducted to find high quality guidelines that were applicable to our patient population [11]. As no guideline was suitable to be used straight ‘off the shelf’, we selected four high-quality and applicable guidelines [28–31] for in-depth content review and adaptation. After establishing an interprofessional guideline adaptation group (consisting of a palliative care physician with a delirium research interest (SHB), nursing practice leader (ES), practice support nurse, pharmacist, social worker, spiritual care provider and unit clinical manager), an interprofessional delirium care pathway was mapped out. This was scaled down into manageable colour-coded domains by team consensus (See Fig. 1). As comprehensive and lengthy guidelines can be challenging to implement, rather than developing a single large module, we developed short modules to facilitate phased implementation with a pragmatic approach. The Nursing Delirium Screening Scale (Nu-DESC) [32] was the focus for the screening and assessment module as it was already in use on our unit for many years but with limited formal structured training for new nurses.

Answers to key health questions regarding the care of patients with delirium were sought by performing a content analysis of the selected guidelines, and creating two recommendation matrices, [6] one each for non-pharmacological and pharmacological management. These were the foundation for our local guideline. An additional recommendations matrix on pharmacological interventions for delirium was created from published evidence-based reviews in cancer and older populations [34–36], but this became redundant once the study by Agar et al. was published [22]. Despite extensive discussions in three physician meetings, there was no consensus on the role of antipsychotics and dosing. Thus, rather than creating rigid pharmacological recommendations, a pharmacological ‘framework’ was created as a critical component of the pharmacological module [37].

### Development of guideline educational content

The guideline adaptation group developed a 45-min introductory interprofessional module, called ‘The Starter Kit’, to be delivered as multiple small group mandatory face-to-face participatory sessions. Our messaging was that while participants may already know and practise much of the guideline content, the difference with this guideline was its systematic approach and incorporation of new emerging evidence on antipsychotics. The nursing practice leader developed an additional 15-min face-to-face module on monitoring for nurses.

The remainder of the guideline content was developed as four online self-learning modules (e-Learning modules): nurse delirium screening, non-pharmacological interventions, communicating with a delirious person, and pharmacological management. (See Table 1 for further details). These modules were created in Microsoft Powerpoint® (2010) [38] with voiceover and uploaded to the hospital’s online learning system, and were to be completed according to the individual’s team role. A family information booklet was created from published literature on the patient and family delirium experience, [39] incorporating input from the interprofessional team and bereaved family members.

**Table 1:** Overview of content of modules and implementation resources for the unit interprofessional delirium guideline

**Title of Module**	**Format** *(Approximate Duration)*	**Intended Audience**	**Notes**
Introductory *interprofessional* module: ‘The Starter Kit’	Mandatory face-to-face training session (IP presenters from the guideline adaptation group) *(45 min)*	**All** healthcare team (including ward clerks, porters) and volunteers	- Designed as fundamental ‘core’ delirium education and also to orientate learners to the content of the guideline and subsequent e-Learning modules - Describes features of delirium, and terminology used - Provides brief contrast of delirium screening tools used by different members of the IP team: Nu-DESC [32] *(used by nurses only)*, CAM [40] *(used by physicians only)*, and SQiD [41] *(new tool for PCU: introduced in order to be used by other members of IP team, PCU staff and volunteers)* - Gives outline of non-pharmacological interventions *(All attendees given copy of patient/family delirium information leaflet)*
Evaluation and monitoring a delirious patient	Mandatory face-to-face training session, at end of ‘Starter Kit’ session (NPL/ PSN presenters) *(15 min)*	Nurses only	- Linked with our organisation’s Nursing “Always Practices”, i.e. Bedside shift report; hourly rounding; update patient care boards; start/end of shift assessments; safety huddle; and priority lists
Nurse-driven delirium screening on the PCU	e-Learning module *(15 min)*	Nurses only	- To review the Nu-DESC tool (a paper version of the tool had been in use on our PCU for many years before this project started but the Nu-DESC had recently been embedded within the EPR as standardised PCU documentation for nursing at the end of each 8-h shift) - Provides detailed information on how to rate the 5 Nu-DESC observed symptoms - Includes 2 case studies (hyperactive vs. hypoactive delirium) for nurses to rate the Nu-DESC and compare results with peers
Delirium care tips: Non-pharmacological strategies for delirium management on the PCU	e-Learning module (with voiceover) *(10 min)*	**All** healthcare team (including ward clerks, and porter)	- Multicomponent interventions derived from the content analysis of pre-existing comprehensive delirium guidelines: patient orientation including use of communication aids (glasses, hearing aids, dentures); optimise sleep–wake pattern; safety of patient environment; avoid unnecessary use of urinary catheters; encourage ADLs; promote safe mobility as tolerated (avoid use of physical restraints); assess and control pain; monitor hydration, nutrition, bowel and bladder function, pressure areas and assess for hypoxia; provide support and education to patient and family - Includes delirium “Care Tips” document as a reminder for nursing staff, accessible as a link in the EPR
Tips for communicating with delirious patients	e-Learning module (with voiceover) *(10 min)*	**All** healthcare team (including ward clerks, and porter)	- Strategies derived from the content analysis of pre-existing comprehensive delirium guidelines, and from published literature on the patient delirium experience - Provides link to the European Delirium Association patient delirium experience teaching video [42]
Pharmacological management of delirium in palliative care	e-Learning module *(30–40 min)*	Physicians, nursing, pharmacist	- Focuses on starting with a targeted approach of ‘as needed’ pharmacological interventions (if non-pharmacological interventions have been ineffective) for distressing delirium perceptual disturbances (e.g. hallucinations, illusions) or if safety concerns, with lower medication doses for older or frail patients - Highlights importance of medication review and deprescribing, and adverse effects of pharmacological interventions, e.g. EPS with APs, avoid haloperidol in patients with Parkinson’s Disease or Dementia with Lewy bodies, possible increased patient agitation and delirium with APs and BDZs - Uses 4 interactive patient cases to illustrate different scenarios - Downloadable prescribing ‘framework’ document (Adapted from [37])
Patient/ family education	Bilingual (English and French) delirium information leaflets[43]	Patients and their families	- Includes signs and symptoms of delirium; how to communicate with a person who has delirium; how the family can help care for the person with delirium
**Implementation resources**	**Format**	**Intended Audience**	**Notes**
(1) Accessible ‘Big Picture summary’ of the modular delirium guideline	2-sided laminated sheet: Page 1: brief overview summary of delirium guideline modules Page 2: summary of pharmacological management for distressing delirium symptoms	Nursing, physicians, pharmacist, medical learners	Inserted at front of all patient binders for medication administration records as a point-of-care tool
(2) Guideline algorithm for the management of delirium in palliative care patients	Wall poster (using same colours as guideline modules)	Clinical staff	Posted in prominent position in each of the PCU charting rooms (Adapted from [1])

Abbreviations: *ADL* Activities of daily living, *AP* Antipsychotic, *BDZ* Benzodiazepine, *CAM* Confusion Assessment Method, *EPR* Electronic patient record, *EPS* Extrapyramidal side-effects, *IP* Interprofessional, *NPL* Nursing practice leader, *Nu-DESC* Nursing Delirium Screening Scale [32], *PCU* Palliative care unit, *PSN* Practice support nurse, *SQiD* Single Question in Delirium

### Implementation planning by guideline adaptation group

Two implementation resources were developed: a practical easy-to-follow coloured ‘Big Picture summary’ and a poster-sized version of a coloured guideline algorithm. (See Table 1 for further details). Planning involved deciding optimal timing for guideline rollout. (See Table 2 for implementation timeline). Hospital management agreed to fund extra nursing coverage whenever nursing staff attended the combined ‘starter kit’ and monitoring sessions during a rostered shift, and nurse education sessions outside of working hours.

**Table 2:** Project timeline for adaptation, implementation, and evaluation of the interprofessional modular delirium clinical practice guideline

DATE	DETAILS
**Initial steps:**	
Autumn 2013	Established initial core guideline group (physician, pharmacist, PSN and RN) – regular meetings; grant submission
February 2014	With PSN (APN position remained vacant), proceed with launch of ‘Starter Kit’ as mini opportunistic ‘lunch and learn’ sessions to nursing in the PCU break room during their lunch breaks
March 2014 – November 2014	Significant PCU staff changes across the PCU team (nursing, PSN, allied health, unit CM) necessitating stop of ‘Starter Kit’ launch in March; PCU and other institution-wide training priorities
December 2014 – February 2015	Develop clinical cases for Nu-DESC module with two experienced PCU RNs; draft non-pharmacological content with experienced PCU RPN; meetings with physicians and pharmacist regarding pharmacological management; change of unit CM
January – April 2015	Hospital EPR rollout
April – June 2015	APN position filled (make tentative plan to launch guideline rollout in July 2015); increased clinical workload on PCU due to increase in number of daily admissions to improve hospital patient flow
June – August 2015	Family delirium information booklet drafted by summer undergraduate medical student: feedback from IP team in focus groups/ interviews
September 2015	New APN (tentative plan to launch guideline rollout in November 2015); PCU and other institution-wide training priorities postpone guideline rollout
October 2015 – March 2016	Clinical lead continues to develop and refine module content
April 2016	APN leaves; Part-time physiotherapist and rehabilitation assistant reassigned to other units
June 2016	New NPL (replacing APN position): initially as 2-month interim position with remit and protected time to focus solely on the delirium CPG project – assists with completion of e-Learning modules
October 2016	Competing institution-wide education projects – guideline implementation deferred
**Full CPG implementation:**	
December 1, 2016	Interprofessional presenter roll-out meeting; All members of GAG given silver-coloured school star badges to be worn on work lanyards so clearly identifiable for the rest of the PCU team
December 5 –15, 2016	Implementation of multiple small group mandatory introductory IP ‘Starter Kit’ sessions over 2 weeks (facilitated by interprofessional presenters from the GAG) in the unit dedicated team rounds room with ‘Evaluation and monitoring’ session component for nursing (facilitated by NPL/PSN); separate ‘Starter Kit’ session held for physicians and medical learners for ease of coordination
December 2016	Launch of 1^st^ 3 e-Learning online modules: - Delirium screening (review of Nu-DESC tool) – *nurses only* - Non-pharmacological strategies – *all staff* - Communication tips with delirious patients – *all staff*
December 2016	Launch of patient and family delirium information leaflet
January 2017	Further ‘Starter Kit’ sessions for nurses and volunteers
June 2017	Pharmacological module and ‘prescribing framework’ finalised
September 5, 2017	Launch of Pharmacological e-Learning online module – *physicians, nurses, pharmacist*
September 5, 2017	Launch of ‘Big Picture’ summary (point-of-care tool)
September 5, 2017	Launch of guideline algorithm
September 5, 2017	Unit celebration event (with CPG colour-coded icing on the celebration cake) on PCU to showcase the work of the GAG. Attended by hospital CEO, senior management, PCU volunteers, staff from across the organisation, and representatives from hospital communications department
December 2017	Deadline for completion of Pharmacological module
**Evaluation:**	
February – April 2018	Pre-CPG patient chart audit of 20 patients admitted in June – December 2015
May 2018	Initial IP team SurveyMonkey® evaluation emails sent out
August 2018	Close of SurveyMonkey®
September 2018 – January 2019	Focus groups/ interviews
October – November 2018	Post-CPG patient chart audit of 20 patients admitted in January – March 2018

Abbreviations: *APN* Advanced practice nurse, *CEO* Chief executive officer, *CM* Clinical manager, *CPG* Clinical practice guideline, *EPR* Electronic patient record, *GAG* Guideline adaptation group, *IP* Interprofessional, *NPL* nursing practice leader, *Nu-DESC* Nursing Delirium Screening Scale [32], *PCU* Palliative care unit, *PSN* Practice support nurse, *RN* Registered nurse, *RPN* Registered practical nurse

### Quality improvement measures

The project process measure was for ≥ 85% of the interprofessional team to complete the guideline modules. Outcome measures were team feedback in respect to the guideline being accessible, practicable and acceptable; reduction in use of antipsychotics in delirious patients; and increase in use of non-pharmacological interventions. Adverse impact on staff workload was used as a balancing measure.

### Evaluation methods for the implemented guideline

**Fig. 2:**
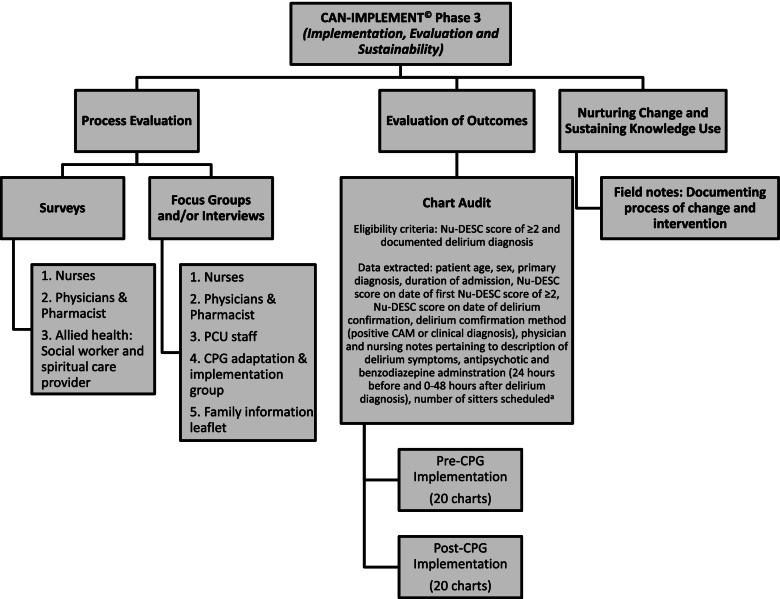
Outline of evaluation strategy for implemented delirium clinical practice guideline based on CAN-IMPLEMENT. © Phase 3 [6]. ^a^Due to change in hospital policy, hospital-paid sitters were not routinely available to sit with patients, so this outcome was not measured. Abbreviations: CAM: Confusion Assessment Method [40]; CPG: clinical practice guideline; IP: interprofessional; Nu-DESC: Nursing Delirium Screening Scale [32]; PCU: palliative care unit

Three evaluation methods assessed the process and impact of guideline implementation: surveys, focus groups/interviews and chart audit (See Fig. 2). The project lead also kept a contemporaneous field journal, recording the project timeline, unanticipated barriers or facilitators to implementation, or other learnings during the process [43].

Three electronic surveys [44] were created for each of the three groups: (1) nurses, (2) physicians and pharmacist, and (3) allied health. Each survey contained 2–4 demographic questions, followed by 20–31 guideline-focused questions specific to team role, and concluded with an open-ended text box. (See Additional file 3 for example of evaluation survey). Survey development was informed by the Smart multi-dimensional model of clinical utility, [45] the Theoretical Domains Framework questionnaire, [7, 8] and an emergency department implementation survey [46]. Invitation and reminder emails to listserve groups of the interprofessional team included a survey link for anonymous and voluntary completion. The beginning of the survey included the participant informed consent form.

Unit staff were invited to participate in semi-structured focus groups or one-on-one interviews. Interview guide questions drew on the overarching project goal of developing a guideline which focused on both process and outcomes of care [47]. (See Additional file 4 for example of interview guide). Questions focused on exploring prior experiences with an emphasis on barriers and facilitators (people, processes) that arose in caring for delirious patients and their family members, understanding how staff engaged with guidelines overall, and participants’ recollection and connection with the delirium guideline module content. Focus groups and interviews were conducted by the research assistant (MK) who was independent of the clinical team. Audio recordings were transcribed verbatim. Transcripts were de-identified and imported into NVivo 12 [48] for analysis.

The retrospective chart audit was conducted by two investigators (SHB, MK). Consecutive charts of patients (by admission date) who were admitted in June – December 2015 and January – March 2018 were screened for documentation of a Nu-DESC score of ≥ 2 (i.e. a positive screen for delirium) and included in the audit if there was a physician-documented delirium diagnosis, either a Confusion Assessment Method (CAM) [40] positive assessment or a clinical diagnosis. Data were extracted onto a standardised password-protected spreadsheet and included pharmacological interventions for delirious patients and clinical care documentation by nurses and physicians. Figure 2 provides further details.

### Analysis

Descriptive statistics were computed for quantitative data (survey and chart audit) using Microsoft Excel® (2010) [49]. Individuals with incomplete survey responses (≤ 50% of survey completed) were excluded from the analysis. Qualitative data were analysed using an iterative, inductive thematic analysis approach [50] by two researchers (MK, MD), both trained in qualitative research methods. In this approach, the researchers independently generated initial (along with in vivo) codes [51] using 30% of the dataset (three transcripts), incorporating language used by participants to remain as close to the data as possible. These initial codes were further refined in analysis meetings between the two researchers, and a codebook was developed with descriptions for each code. Each researcher independently coded the remainder of the dataset using this codebook. Following the flexible approach of thematic analysis, [50] iterative changes were made to the codebook throughout analysis. Codes were aggregated into potential overarching themes and subthemes based on the researchers’ interpretations of the coded excerpts. Themes were further refined throughout analysis, while maintaining coherency across excerpts coded within themes as well as throughout the full dataset [50]. To maintain the rigour and trustworthiness of the analytic approach, two researchers were involved in coding and theme development to facilitate consistency in data interpretation between the researchers and to allow for coding discrepancies to be resolved through discussion. In addition, the data and final analysis were presented to the project lead (SHB), who was not involved in data collection and analysis processes but was familiar with the phenomenon being explored, to validate the findings.

## Results

### Evolution of guideline implementation

The rollout of the ‘starter kit’ was first attempted in February 2014. Implementation stalled due to unexpected major staff changes on the unit and the lack of consistent advanced nursing practice leadership. During this hiatus, work started on the information booklet for families. A shorter bilingual (English and French) ‘patient and family delirium information leaflet’ version was created in time for implementation, with printed copies made available on the unit as well as accessible on the hospital external website [52]. Table 2 shows the detailed project timeline.

Full guideline implementation (of the six modules and additional implementation resources) took 12 months, from December 2016 – December 2017 (See Table 2). The introductory ‘starter kit’ was implemented as 23 face-to-face sessions over two weeks. Sixty-one participants, consisting of unit staff (nurses, physicians, medical learners, allied health, ward clerks and porter), nursing students and instructors, and rostered volunteers, attended with a maximum of ten participants per session. A further seven sessions were delivered to both nurses who missed the rollout and over 30 volunteers as part of their training day.

Three online e-Learning modules were launched in December 2016. The final pharmacological online module was launched in September 2017, with reminder email notification running until December 2017. Overall online module completion rate was 80.4%: delirium screening 73%; non-pharmacological 90%; communication 88.5%; pharmacological 70% (nurses: 74.2% (*n* = 49/66), physicians: 66.6% (*n* = 6/9)).

### Evaluation

#### Survey

**Fig. 3:**
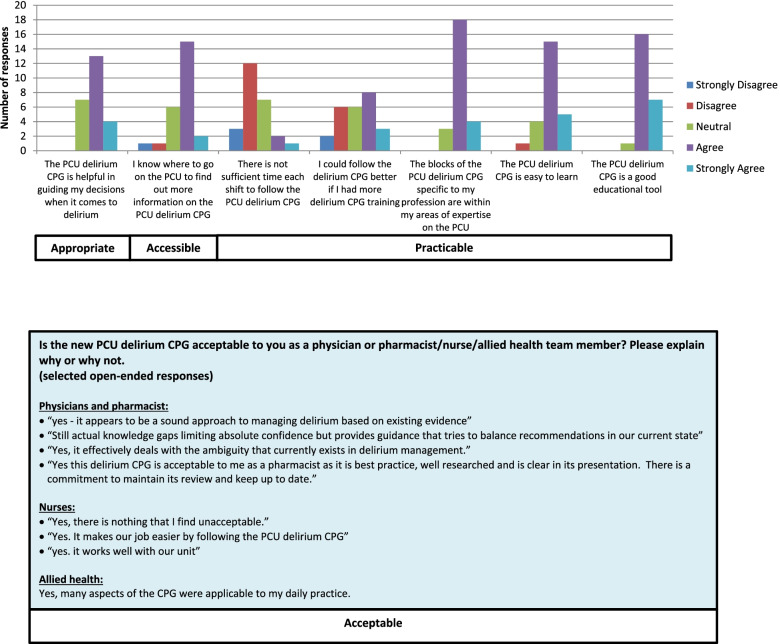
Evaluation survey results: summary of responses based on Smart Model of Clinical Utility [45]. Abbreviations: CPG: clinical practice guideline; PCU: palliative care unit

**Fig. 4:**
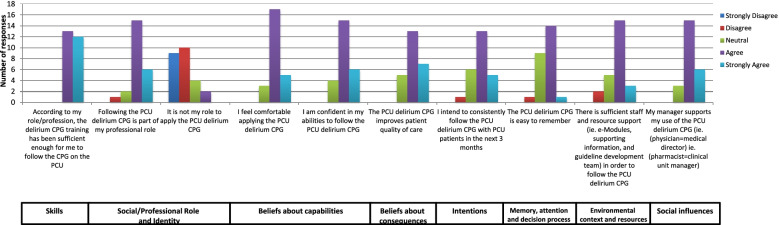
Evaluation survey results: summary of responses by Theoretical Domain Framework [7, 8, 53]. Abbreviations: CPG: clinical practice guideline; PCU: palliative care unit

The overall survey response rate was 32% (25/77 interprofessional team members). (See Additional file 5, Table 1 for respondent demographics). All respondents either strongly agreed (12/25; 48%) or agreed (13/25; 52%) that the training was sufficient for them to follow the guideline in daily practice. Seventeen participants agreed that the guideline was accessible, and 17 participants agreed that it was helpful in guiding their delirium management decisions. Most participants (18/25; 72%) indicated that they intended to consistently follow the guideline in the next three months. Selected survey responses are presented by Smart Model of Clinical Utility in Fig. 3 and by Theoretical Domains Framework in Fig. 4.

#### Chart audit

 Of 75 patient charts screened, 40 with documented evidence of a delirium diagnosis were reviewed: 20 were pre- and 20 were post-guideline implementation. (Patient demographics are presented in Additional file 5, Table 2). Nursing and physician/medical learner documentation of delirium behaviours occurred in 16/20 patient charts pre-guideline implementation and in 17/20 charts post-guideline implementation. Post-guideline implementation, there were four occurrences of nursing documentation of first-line use of non-pharmacological interventions for relief of delirium symptoms or delirium-related distress, compared with none pre-guideline, as shown by these selected quotes:

“Registered Practical Nurse: Was easily calmed when staying with her and talking to her (January 7). Registered Nurse: Continue with nonpharmacological approach as much as possible (January 9). Night nurse: Writer able to effectively settle pt [patient] without pharmacological interventions (January 10)”. [Chart ID C45].

“Night nurse: Pt [patient] rang for nurse—when writer entered room, pt stated he “had to get to [name of nearby town]”. Writer reoriented to place—pt then questioned “where are we now” and when writer stated Ottawa, pt responded, “well I need a transfer then, I need to get back to [name of nearby town]”. Writer redirected and pt able to settle effectively”. [Chart ID C51].

In the 48-h period after a delirium diagnosis, there was approximately 60% less scheduled antipsychotic use, similar ‘as needed’ antipsychotic use, and a 50% increase in ‘as needed’ subcutaneous midazolam administration post-guideline implementation. Additional file 5, Tables 3 and 4 show chart audit results with respect to antipsychotic and benzodiazepine medication administration before and after guideline implementation. See Table 2 for evaluation timeline.

#### Focus groups/ interviews

A total of 10/77 (13%) of palliative care staff participated in focus groups or interviews. The mean length of the two focus groups and three interviews was 19.5 min.

Six key themes were generated from the data (Table 3). Participant perceptions of guideline implementation reflected the temporal nature of changing practice. Specifically, changing practice involved incorporating prior knowledge or experience, confronting challenges during the change, and sustaining change. For some participants, their prior knowledge and extensive experiences of delirium informed their current practices, contributing to viewing the guideline as either basic or supplemental to past experiences. During the implementation process and corresponding changes to current practice, possible challenges to change were identified (e.g., limited staff presence at night). However, these obstacles were mostly seen as surmountable. Guideline implementation reached into participants’ future practice, wherein participants noted elements of their future practice improving, or conversely, not anticipating changes but instead reinforcement of their current practices. Participants also identified particularly significant or helpful elements of the guideline, positing that the guideline was beneficial in providing a common framework and language for the whole team to share. Overall, making changes to current practice was perceived to be a collaborative effort, enabling contributions from unit staff, volunteers, and family caregivers to effectively recognize and manage delirium.

**Table 3:** Qualitative analysis of guideline evaluation interviews and focus groups with staff: key themes and subthemes

Key themes	Subtheme	Description	Notable quotes
**Personal or prior knowledge and experiences that may impact practices with respect to delirium**		This theme included knowledge or experiences from prior to the implementation of the guideline, including from working in a palliative care context for a length of time. This previous knowledge or experience also included practices that the participant already put into use or guidelines they were already familiar with	Physician 1: *“It’s useful for an introductory for people coming from clinical background where they may not be as familiar with delirium, but for those of us who have a fair bit of experience, I think that section definitely files under the basic or introductory category”* Nurse 4: *“I think what happens is we also learned by experience. From each case to each case because every single patient is unique, here, and every single patient reacts differently to medication and there’s family dynamics and the whole thing. So as much as you may have learned back a few years ago when we did the modules, it’s an ongoing learning process, and I think when you take that into consideration, your experience is what’s going to teach you how to as well, even though you have the base, you have the education that’s there”*
**Challenges of facilitating change through implementation of the guideline**		Any difficulties encountered during the implementation of the guideline and/or management of delirium	
	Situational factors	Some challenges experienced by participants involved specific situations impacting guideline implementation and training, such as reduced staff presence at night-time, symptoms being difficult to manage, and different hierarchical or corporate priorities	Nurse 1: *“So then what if you get a confused patient who’s agitated and wants to get out of bed or they want us to get them out of bed, which is no problem during the day, but how’s night shift supposed to do that?”* Physician 2: *“In theory, we all really want to try and manage if we can any episode of delirium with a non-pharmacological approach, but the reality is that we find, not infrequently, we find situations where patients are extremely distressed, agitated. Families are very distressed seeing that, and there was a compelling need for us to intervene more pharmacologically. But yet our knowledge of how we are to best intervene pharmacologically is limited. So that makes that very difficult”*
	Sustaining change	In other cases, participants shared difficulties associated with sustaining continued use of the guideline. Ensuring that practice changes were sustained over time involved the incorporation of the guideline as a reminder, keeping guideline elements at the forefront of care, and changing practices at a pace that enabled uptake	Guideline adaptation group member 1: *“And also being attentive to, from a nursing side of things the pace of change, as well, because in the last few years there have been a lot of changes to bedside nursing practice. Like when you think of all the electronic documentation that’s relatively new, and so it’s making sure that when we’re bringing changes that it can be at a pace that’s absorbed and supported, as well”*
	Time	Other challenges and barriers to implementation of the guideline involved staff time or the length of time since completion of guideline training. Protected staff time to complete the guideline training was described as a method of overcoming this challenge	Guideline adaptation group member 1: *“There was a coordination piece to make sure that we had protected time for staff to attend… for the online modules, for staff who were already on the unit, they were asked to do it on their own time, which—that can account for why people may not have done it, if you’re continuously really busy with patients. But over time, integrated that into protected time within the unit orientation for nurses, as a way to say, you have paid time to learn and understand this stuff”*
**Impacts on practice post-implementation**		Any specific things participants noted have or have not changed in their own practice or in their observations of others’ practices as a result of the guideline training and implementation; includes considerations for future practice	
	Changes in practice	Participants described elements that changed and/or improved, such as increased delirium screening proficiency, better communication, and facilitation of delirium prevention strategies	Physician 1: *“There’ve been a few situations where I’ve really noticed the nurses do things like be descriptive around filling out the Nu-DESC. Maybe they’ll write a note about why they rated highly on certain features. Or I’ve just seen that in certain cases they now will actually rate a very high Nu-DESC quite appropriately, where in the past I hadn’t seen that done… more often now I’ve seen a bit better description of this is the behaviour, medication was given, this is how long it took to work and this is how the behaviour changed. So I almost never saw those descriptions before and now I’m starting to see them…”*
	No changes in practice	Participants also reported elements that had not changed as a result of the guideline. The guideline was seen as a reinforcement of practice elements that were already being done	Physician 1: *“My impression was that maybe it [the guideline] wasn’t really adding new information for the physicians or trying to change their practice. It was just reinforcing what we’re already doing”*
**Collaborative effort of change**		Participants described the team approach to identifying areas for improvement and implementing change in practices for delirium prevention and management	Staff member 1: *“…it [the guideline] works in non-nursing, it increases something, like we communicate with the nurse, with the doctor, so, yeah. It’s a thing where it can actually prevent this especially for evening and at night, if we can protect them during the day time so this evening can have easy going on, same things for nights”* Guideline adaptation group member 1: *“I think having a role of professional nursing leadership on the unit where the role is certainly to support evidence-based practice best practices as it’s for that unit I think are certainly elements that are needed and obviously the involvement of the inter-professional team. And in terms of the clinical manager being immensely supportive, in terms of finding financial resources and [being] emotionally invested and supportive as well, I mean, certainly everyone plays a part in that, but I think it takes champions in different pockets to really make things work and certainly from more upper management, as well, from the directors as well… The other piece that has been important is also having the starter kit integrated into the education for volunteers who are going to be on the Palliative Care Unit. So that’s an important enabler in terms of the inter-professional education”*
**General importance of having standardised guidelines**		Participants conveyed the general benefits of having standardised guidelines as a reference as part of clinical or teaching practices. These benefits included having a common language or reference point for delirium management across the whole PCU team	Physician 1: *“I find for the most part it [the guideline] becomes part of our habitual practice and then we’re not using them on a case-by-case and day-by-day practice, but they’re useful for those instances where you do just to refer back. Sometimes useful from a teaching perspective, as well… And, lastly, I think, just in terms of practice standardization for the interaction between physicians and nurses and understanding when we talk about whether it’s palliative sedation or delirium or a bowel protocol, that we all have a similar framework or reference point, and we’re talking the same language”*
**Elements of guideline or guideline training that individual found important, helpful, or useful**		Participants also noted items or aspects of the delirium guideline or provided training that were important or helpful to caring for someone with delirium, including: i) the ‘Big Picture’ summary format of the guideline; ii) multi-module nature and user-friendliness; iii) helpfulness in increasing knowledge for non-nursing PCU staff	Guideline adaptation group member 1: *“In terms of design and consideration to the accessibility of the guidelines, I think it’s really excellent because it’s very user-friendly. It’s also, in terms of being able to think about clinical problems overall with a similar approach, I think that that’s really thought out. It’s accessible in terms of having a variety of learning strategies and ways to support practices on the unit, so it’s multi-modal in terms of in-class time, online modules, having components that are visual, having it reinforce with things in the MAR. So it’s accessible on different fronts, so I think that’s really positive, and I think it also really ties in—and, again, I’m speaking from a nursing side of things—but to really tie into really practical examples of what nurses encounter in their clinical practice into the role of the guidelines I think is a very important piece”*

Abbreviations: *MAR* Medication administration record; *PCU* Palliative care unit

### Unexpected benefits of implementation process

From the field journal, an unexpected benefit of pivoting to the last component (family information booklet) was that it really engaged the team, encouraged thinking about delirium, and gave them ownership of the resource, as it incorporated their feedback. The face-to-face small group sessions enabled presenters to gauge the current knowledge of the attending participants. If participants demonstrated good fundamental knowledge of delirium, then more content and discussion was added to their specific session, making it a dynamic process that was flexible to participants’ needs. Field notes also captured team members’ personal comments and observed practice changes during guideline implementation.

## Discussion

Our guideline was adapted to meet our local context in order to encourage increased acceptance [54]. This project confirmed that the process of guideline adaptation can lead to engagement and capacity building, with a participatory approach developing a ‘community of practice’ [25]. Although this was the first time that interprofessional team members had worked together on such a project and regular meetings of the guideline adaptation group were a significant time commitment, the experience has helped shape our ongoing implementation and quality improvement work as a team.

We used multimodal education interventions with narratives [55], rather than solely relying on printed or electronic educational materials for guideline dissemination [56], to implement a novel modular guideline to the entire interprofessional team, including staff and volunteers, to improve fundamental delirium care on our palliative care unit. As successful guideline implementation typically requires behaviour change from more than a single professional group, [47] an intentional interprofessional guideline embraces and targets all groups simultaneously. While our interprofessional guideline domains (module topics) had been developed by consensus, we later discovered that they aligned with the quality statements of the 2014 National Institute for Health and Care Excellence (NICE) Quality Standard for delirium [57].

Consistent with CAN-IMPLEMENT©, [6] our guideline adaptation and implementation process was non-linear, dynamic and iterative. It can take an average of 17 years before research evidence changes clinical practice, [58] but our project enabled the timely incorporation of recent evidence [22] into bedside management. By presenting pharmacological interventions as a ‘framework’ rather than a prescriptive guideline on prescribing that removes physician autonomy, [59] it may be possible to nudge practice change. However, while our guideline recommended the prescribing of medications in lower doses than previously used and on an ‘as needed’ basis (as opposed to scheduled dosing), further research on the role of antipsychotics and benzodiazepines in delirium management is still needed [60, 61]. Follow up Plan-Do-Study-Act cycles will be needed to assess physician prescribing practices on an ongoing basis.

Several organisational contextual factors challenged the development of e-Learning modules and establishing rollout plans, with the final guideline implementation taking 12 months. While our implementation facilitators (including relevance to our patient population, management support, development of ‘user-friendly’ education resources, enthusiastic ‘delirium champions’, and a new patient education resource) were as previously reported, [62–64] our project also demonstrated the vital consideration of contextual factors for successful guideline implementation. [65, 66] These included the need for stability within the project team, advanced nursing practice leadership, protected time, and financial support for nurse attendance at education sessions [63, 67–69].

Although the eventual ‘starter kit’ implementation was time- and resource-intensive, it generated momentum for the remaining modules. While 100% of the interprofessional team participated in a ‘starter kit’ session, the completion rate for the subsequent four e-Learning modules varied from 70–90%. Our pharmacological module had the lowest completion rate. This may have been due to its length (as completion occurred during work hours or personal time) or staff reasoning that they already had the necessary requisite knowledge. In the future, to improve compliance with e-Learning module completion, dedicated time for staff completion should be considered. With an overall completion rate for all five modules of 90.2%, our reach was higher than previously reported in a trial of delirium guideline implementation in medical inpatients where overall attendance by ward staff to five 30-min topic education sessions was 40–73% [70]. As part of our guideline sustainability efforts, all new hired nursing staff receive the ‘starter kit’ session and are required to complete the e-Learning modules as part of onboarding. The ‘starter kit’ module is embedded in the orientation of new volunteers and a new 1-h lunchtime teaching session incorporating the elements of the delirium guideline is delivered monthly to rotating medical learners. Long-term management support may become challenging given the current fiscal environment. More research is needed on updating content and guideline sustainability with the implementation of periodically updated modules.

From the evaluation, surveyed participants all ‘agreed’ or ‘strongly agreed’ that the training had been sufficient. Interviewed participants confirmed that the collaborative guideline improved or reinforced their current practices and provided a common framework and language that could be used by the whole team [71]. Scheduled antipsychotic use declined. However, despite the historical avoidance of benzodiazepines in delirium, [1] there was proportionally more use of ‘as needed’ midazolam post-guideline implementation. In addition to studies examining non-pharmacological interventions and the role of pharmacological agents in the prevention of delirium and management of delirium symptoms in this patient population, further research is needed on patient-reported outcomes for the relief of delirium symptoms and associated distress.

### Strengths and limitations

A strength of this project was the involvement of interprofessional team members in the guideline adaptation group at inception and the framework approach. Multiple evaluation methods added rigour. Qualitative methods were a notable component to capture the complex relationship between implementation processes, places and people [72].

A study limitation includes implementation in a single centre with its own unique culture. Different delirium guideline adaptation and implementation processes may be required for other settings and countries. Our project also had a much longer than anticipated lead-in phase, but the provision of two months of dedicated nursing practice leader time proved to be essential in enabling full guideline implementation. Although we followed the steps of the CAN-IMPLEMENT© Version 3.0 resource, [6, 25] the scalability of the utilised approach is unknown, especially given our demonstrated need for significant team and management support. Chart auditors were not blinded as to the purpose of the project, and it is possible that the prescribing practice of some physicians had started to change before implementation of the pharmacological module. We did not examine adherence or recording of ‘dose’ of multicomponent non-pharmacological interventions for delirium and further research is needed in this area [73]. Despite reminder emails, the response rate to the evaluation survey was low. Allocated time for staff completion of surveys may improve completion rates in the future. While the Guideline Development Groups for the four high-quality delirium guidelines [28–31] that we adapted had included patient members, we did not examine the acceptability of the delirium guideline from the patient/family caregiver perspective. Bereaved family caregivers had been interviewed as part of the development of our draft delirium family information booklet to capture their recommendations from their lived experience on the palliative care unit [74]. Future iterations of the palliative care unit delirium guideline would benefit from co-production with patient and family involvement as local project partners [75].

## Conclusion

Although clinical practice guidelines can provide consistency in evidence-informed collaborative interprofessional practice, guideline adaptation and the development of supporting education sessions takes significant time and effort, requiring management support, nursing leadership, and interprofessional champions with protected time. Guideline implementation requires an agile and flexible team. Our report of the implementation of a novel modular guideline to an entire inpatient interprofessional team, including non-clinical staff and volunteers, using an education initiative, provides useful insights for other teams undertaking guideline implementation. Post-implementation evaluation is a critical component to demonstrate impact, both clinically and on the team. Future research should examine the sustainability of guidelines in palliative care settings and scaling up for multisite implementation.

## Supplementary Information

**Additional file 1.** Completed Standards for Quality Improvement Reporting Excellence (SQUIRE) 2.0 checklist. Provides manuscript location for checklist items.

**Additional file 2.** Completed Template for Intervention Description and Replication (TIDieR) checklist. Provides manuscript location for checklist items.

**Additional file 3.** Delirium Clinical Practice Guideline Survey for Palliative Care Unit (PCU) nurses. Evaluation survey administered to Palliative Care Unit nurses.

**Additional file 4.** Delirium clinical practice guideline focus group/interview guide for Palliative Care Unit (PCU) nurses. Focus group/interview guide used with Palliative Care Unit nurses in interviews to understand their experiences of using the delirium clinical practice guideline.

**Additional file 5:** **Compiled Supplementary Tables:** Additional results for the delirium clinical practice guideline evaluation survey, and pre- and post-implementation chart audit. **Table 1.** Respondent demographics for evaluation survey. **Table 2.** Patient demographics for pre- and post- delirium guideline implementation chart audit. **Table 3.** Chart audit results for antipsychotic and benzodiazepine medication administration 24 hours before documented delirium diagnosis. **Table 4.** Chart audit results for antipsychotic and benzodiazepine medication administration 0-48 hours after documented delirium diagnosis.

## Data Availability

The study data are held at the Bruyère Research Institute. The datasets used and analysed during the current study are available from the corresponding author on reasonable request. To preserve the anonymity of the interviewees, the transcribed interviews are not available for sharing.

## Abbreviations

CAM: Confusion Assessment Method
NICE: National Institute for Health and Care Excellence
Nu-DESC: Nursing Delirium Screening Scale
SQUIRE: Standards for Quality Improvement Reporting Excellence
TIDieR: Template for Intervention Description and Replication
