# Supplementary material for: Adaptation, implementation, and mixed methods evaluation of an interprofessional modular clinical practice guideline for delirium management on an inpatient palliative care unit
Source: BMC Palliat Care. 2022 Jul 16;21:128. doi: 10.1186/s12904-022-01010-6 (PMC9287908; doi:10.1186/s12904-022-01010-6)
Supplement: Supplementary file 1 — Additional file 1. Completed Standards for Quality Improvement Reporting Excellence (SQUIRE) 2.0 checklist. Provides manuscript location for checklist items. [file 12904_2022_1010_MOESM1_ESM.docx]

| **Revised Standards for Quality Improvement Reporting Excellence (SQUIRE 2.0) September 15, 2015** | | |
| --- | --- | --- |
| **Text Section and Item Name** | **Section or Item Description** |  |
| **Notes to authors** | - The SQUIRE guidelines provide a framework for reporting new knowledge about how to improve healthcare - The SQUIRE guidelines are intended for reports that describe system level work to improve the quality, safety, and value of healthcare, and used methods to establish that observed outcomes were due to the intervention(s). - A range of approaches exists for improving healthcare. SQUIRE may be adapted for reporting any of these. - Authors should consider every SQUIRE item, but it may be inappropriate or unnecessary to include every SQUIRE element in a particular manuscript. - The SQUIRE Glossary contains definitions of many of the key words in SQUIRE. - The Explanation and Elaboration document provides specific examples of well-written SQUIRE items, and an in-depth explanation of each item. - Please cite SQUIRE when it is used to write a manuscript. | **As you review the manuscript, place a checkmark in this column for each SQUIRE item that is appropriately addressed in the manuscript.**  **Remember that not every item is necessary in every manuscript.** |
| **Title and Abstract** |  |  |
| **1. Title** | Indicate that the manuscript concerns an initiative to improve healthcare (broadly defined to include the quality, safety, effectiveness, patient-centeredness, timeliness, cost, efficiency, and equity of healthcare) | N/A (Guideline implementation rather than formal QI project) |
| **2. Abstract** | 1. Provide adequate information to aid in searching and indexing 2. Summarize all key information from various sections of the text using the abstract format of the intended publication or a structured summary such as: background, local problem, methods, interventions, results, conclusions | Page 6-7 |

| **Introduction** | *Why did you start?* |  |
| --- | --- | --- |
| **3. Problem Description** | Nature and significance of the local problem | Page 9 |
| **4. Available knowledge** | Summary of what is currently known about the problem, including relevant previous studies | Page 8-9 |
| **5. Rationale** | Informal or formal frameworks, models, concepts, and/or theories used to explain the problem, any reasons or assumptions that were used to develop the intervention(s), and reasons why the intervention(s) was expected to work | Page 11 |
| **6. Specific aims** | Purpose of the project and of this report | Page 10 |
| **Methods** | *What did you do?* |  |
| **7. Context** | Contextual elements considered important at the outset of introducing the intervention(s) | Page 10 |
| **8. Intervention(s)** | 1. Description of the intervention(s) in sufficient detail that others could reproduce it 2. Specifics of the team involved in the work | Page 11-13  Figure 1;Table 1  Table |
| **9. Study of the Intervention(s)** | 1. Approach chosen for assessing the impact of the intervention(s) 2. Approach used to establish whether the observed outcomes were due to the intervention(s) | Page 14-16 |
| **10. Measures** | 1. Measures chosen for studying processes and outcomes of the intervention(s), including rationale for choosing them, their operational definitions, and their validity and reliability 2. Description of the approach to the ongoing assessment of contextual elements that contributed to the success, failure, efficiency, and cost 3. Methods employed for assessing completeness and accuracy of data | Page 14  Figure 2 |
| **11. Analysis** | 1. Qualitative and quantitative methods used to draw inferences from the data 2. Methods for understanding variation within the data, including the effects of time as a variable | Page 16-17 |
| **12. Ethical**  **Considerations** | Ethical aspects of implementing and studying the intervention(s) and how they were addressed, including, but not limited to, formal ethics review and potential conflict(s) of interest | Page 10 |

| **Results** | *What did you find?* |  |
| --- | --- | --- |
| **13. Results** | 1. Initial steps of the intervention(s) and their evolution over time (*e.g.*, time-line diagram, flow chart, or table), including modifications made to the intervention during the project 2. Details of the process measures and outcome 3. Contextual elements that interacted with the intervention(s) 4. Observed associations between outcomes, interventions, and relevant contextual elements 5. Unintended consequences such as unexpected benefits, problems, failures, or costs associated with the intervention(s). 6. Details about missing data | Page 17-21;  Table 2;  Fig 3; Fig 4; Table 7  Additional file 5 |
| **Discussion** | *What does it mean?* |  |
| **14. Summary** | 1. Key findings, including relevance to the rationale and specific aims 2. Particular strengths of the project | Page 21-24 |
| **15. Interpretation** | 1. Nature of the association between the intervention(s) and the outcomes 2. Comparison of results with findings from other publications 3. Impact of the project on people and systems 4. Reasons for any differences between observed and anticipated outcomes, including the influence of context 5. Costs and strategic trade-offs, including opportunity costs | Page 21-24 |
| **16. Limitations** | 1. Limits to the generalizability of the work 2. Factors that might have limited internal validity such as confounding, bias, or imprecision in the design, methods, measurement, or analysis 3. Efforts made to minimize and adjust for limitations | Page 24-25 |
| **17. Conclusions** | 1. Usefulness of the work 2. Sustainability 3. Potential for spread to other contexts 4. Implications for practice and for further study in the field 5. Suggested next steps | Page 25 |
| **Other information** |  |  |
| **18. Funding** | Sources of funding that supported this work. Role, if any, of the funding organization in the design, implementation, interpretation, and reporting | Page 27 |
