# Supplementary material for: Adaptation, implementation, and mixed methods evaluation of an interprofessional modular clinical practice guideline for delirium management on an inpatient palliative care unit
Source: BMC Palliat Care. 2022 Jul 16;21:128. doi: 10.1186/s12904-022-01010-6 (PMC9287908; doi:10.1186/s12904-022-01010-6)
Supplement: Supplementary file 3 — Additional file 3. Delirium Clinical Practice Guideline Survey for Palliative Care Unit (PCU) nurses. Evaluation survey administered to Palliative Care Unit nurses. [file 12904_2022_1010_MOESM3_ESM.docx]

Supplementary File 1: Delirium Clinical Practice Guideline Survey for Palliative Care Unit (PCU) nurses.

# Question 1

What is your role on the Palliative Care Unit (PCU)?

|  | Registered Nurse (RN) |
| --- | --- |
|  | Registered Practical Nurse (RPN) |
|  | Advanced Practice Registered Nurse (APN) |
|  | Other, please specify... ______________________ |

# Question 2

How long have you worked on the Elisabeth Bruyère PCU?

|  | <5 years |
| --- | --- |
|  | 5-10 years |
|  | 10-15 years |
|  | >15 years |
|  | Please specify number of years worked elsewhere in palliative care ______________________ |

# Question 3

What is your employment status on the PCU?

|  | Full-time regular PCU staff |
| --- | --- |
|  | Part-time regular PCU staff |
|  | non-regular PCU staff |
|  | Other, please specify... ______________________ |

# Question 4

What shift do you primarily work?

|  | Day |
| --- | --- |
|  | Evening |
|  | Night |

# Question 5

Are you familiar with the PCU delirium clinical practice guideline (CPG)?

|  | Yes |
| --- | --- |
|  | No |

# Question 6

Which of the following training sessions/e-Modules surrounding the PCU delirium CPG did you attend/complete?

|  | Starter Kit (face to face training session) |
| --- | --- |
|  | Nu-DESC review for PCU Nurses (e-Module) |
|  | Non-pharmacological Strategies (e-Module) |
|  | Communication Tips (for communication with delirious patients) (e-Module) |
|  | Pharmacological Management (e-Module) |
|  | Evaluation and Monitoring – Using the RASS-PAL (done as face to face training session after Starter Kit session) |
|  | Delirium Information leaflet for patients and their families |
|  | Other, please specify... ______________________ |

# The following questions are scale-based questions. Please check one box for each question.

1=Strongly Disagree..........2=Disagree..........3=Neutral..........4=Agree..........5=Strongly Agree

# Question 7

|  | 1 | 2 | 3 | 4 | 5 |
| --- | --- | --- | --- | --- | --- |
| The PCU is used to changing practices/procedures based on new evidence |  |  |  |  |  |

# Question 8

|  | 1 | 2 | 3 | 4 | 5 |
| --- | --- | --- | --- | --- | --- |
| According to my role/profession, the delirium CPG training has been sufficient enough for me to follow the CPG on the PCU |  |  |  |  |  |

# Question 9

|  | 1 | 2 | 3 | 4 | 5 |
| --- | --- | --- | --- | --- | --- |
| I could follow the delirium CPG better if I had more delirium CPG training |  |  |  |  |  |

# Question 10

|  | 1 | 2 | 3 | 4 | 5 |
| --- | --- | --- | --- | --- | --- |
| The PCU delirium CPG is easy to learn |  |  |  |  |  |

# The following questions are scale-based questions. Please check one box for each question.

1=Strongly Disagree..........2=Disagree..........3=Neutral..........4=Agree..........5=Strongly Agree

# Question 11

|  | 1 | 2 | 3 | 4 | 5 |
| --- | --- | --- | --- | --- | --- |
| The PCU delirium CPG is a good educational tool |  |  |  |  |  |

# Question 12

|  | 1 | 2 | 3 | 4 | 5 |
| --- | --- | --- | --- | --- | --- |
| The PCU delirium CPG is a convenient source of advice |  |  |  |  |  |

# Question 13

|  | 1 | 2 | 3 | 4 | 5 |
| --- | --- | --- | --- | --- | --- |
| I feel comfortable applying the PCU delirium CPG |  |  |  |  |  |

# Question 14

|  | 1 | 2 | 3 | 4 | 5 |
| --- | --- | --- | --- | --- | --- |
| The PCU delirium CPG is an efficient use of my time |  |  |  |  |  |

# Question 15

|  | 1 | 2 | 3 | 4 | 5 |
| --- | --- | --- | --- | --- | --- |
| The use of PCU delirium CPG is beneficial to my practice |  |  |  |  |  |

# The following questions are scale-based questions. Please check one box for each question.

1=Strongly Disagree..........2=Disagree..........3=Neutral..........4=Agree..........5=Strongly Agree

# Question 16

|  | 1 | 2 | 3 | 4 | 5 |
| --- | --- | --- | --- | --- | --- |
| The PCU delirium CPG is easy to use |  |  |  |  |  |

# Question 17

|  | 1 | 2 | 3 | 4 | 5 |
| --- | --- | --- | --- | --- | --- |
| The PCU delirium CPG is easy to remember |  |  |  |  |  |

# Question 18

|  | 1 | 2 | 3 | 4 | 5 |
| --- | --- | --- | --- | --- | --- |
| The PCU delirium CPG improves patient quality of care |  |  |  |  |  |

# Question 19

|  | 1 | 2 | 3 | 4 | 5 |
| --- | --- | --- | --- | --- | --- |
| Following the PCU delirium CPG is part of my professional role |  |  |  |  |  |

# Question 20

|  | 1 | 2 | 3 | 4 | 5 |
| --- | --- | --- | --- | --- | --- |
| I know where to go on the PCU/Bruyère eLearning system to find out more information about the PCU delirium CPG |  |  |  |  |  |

# The following questions are scale-based questions. Please check one box for each question.

1=Strongly Disagree..........2=Disagree..........3=Neutral..........4=Agree..........5=Strongly Agree

# Question 21

|  | 1 | 2 | 3 | 4 | 5 |
| --- | --- | --- | --- | --- | --- |
| The clinical unit manager supports my use of the PCU delirium CPG |  |  |  |  |  |

# Question 22

|  | 1 | 2 | 3 | 4 | 5 |
| --- | --- | --- | --- | --- | --- |
| There are nurse role models/leaders in my unit that advocate the use of the PCU delirium CPG |  |  |  |  |  |

# Question 23

|  | 1 | 2 | 3 | 4 | 5 |
| --- | --- | --- | --- | --- | --- |
| There will be fewer complaints from our patients/families when we apply the PCU delirium CPG |  |  |  |  |  |

# Question 24

|  | 1 | 2 | 3 | 4 | 5 |
| --- | --- | --- | --- | --- | --- |
| The PCU delirium CPG is helpful in guiding my decisions when it comes to delirium |  |  |  |  |  |

# Question 25

|  | 1 | 2 | 3 | 4 | 5 |
| --- | --- | --- | --- | --- | --- |
| There is sufficient staff and resource support to be able to follow the PCU delirium CPG |  |  |  |  |  |

# The following questions are scale-based questions. Please check one box for each question.

1=Strongly Disagree..........2=Disagree..........3=Neutral..........4=Agree..........5=Strongly Agree

# Question 26

|  | 1 | 2 | 3 | 4 | 5 |
| --- | --- | --- | --- | --- | --- |
| The blocks of the PCU delirium CPG specific to my profession are within my areas of expertise on the PCU |  |  |  |  |  |

# Question 27

|  | 1 | 2 | 3 | 4 | 5 |
| --- | --- | --- | --- | --- | --- |
| I am confident in my abilities to follow the PCU delirium CPG |  |  |  |  |  |

# Question 28

|  | 1 | 2 | 3 | 4 | 5 |
| --- | --- | --- | --- | --- | --- |
| It is not the nurses’ role to apply the PCU delirium CPG |  |  |  |  |  |

# Question 29

|  | 1 | 2 | 3 | 4 | 5 |
| --- | --- | --- | --- | --- | --- |
| There is not sufficient time each shift to follow the PCU delirium CPG |  |  |  |  |  |

# Question 30

|  | 1 | 2 | 3 | 4 | 5 |
| --- | --- | --- | --- | --- | --- |
| The PCU delirium CPG is too rigid to apply to individual patients |  |  |  |  |  |

# Question 31

|  | 1 | 2 | 3 | 4 | 5 |
| --- | --- | --- | --- | --- | --- |
| I intend to consistently follow the PCU delirium CPG with PCU patients in the next 3 months |  |  |  |  |  |

# The following questions are text-based questions. Please answer them to the best of your ability.

# Question 32

Based on your role/profession, please describe the central elements of the PCU delirium CPG that you integrate into your practice.

# Question 33

Is the new PCU delirium CPG practical? Please explain why or why not

# Question 34

In your opinion, has the new PCU delirium CPG been effective? Please explain why or why not

# Question 35

Is the new PCU delirium CPG acceptable to you as a nurse? Please explain why or why not

# Question 36

Do you have any other comments or concerns surrounding the PCU delirium CPG?

# Please note that once you click submit, the data used from this survey will no longer be able to be withdrawn from the study.

# Thank you for your time in answering this survey
