# Supplementary material for: Adaptation, implementation, and mixed methods evaluation of an interprofessional modular clinical practice guideline for delirium management on an inpatient palliative care unit
Source: BMC Palliat Care. 2022 Jul 16;21:128. doi: 10.1186/s12904-022-01010-6 (PMC9287908; doi:10.1186/s12904-022-01010-6)
Supplement: Supplementary file 4 — Additional file 4. Delirium clinical practice guideline focus group/interview guide for Palliative Care Unit (PCU) nurses. Focus group/interview guide used with Palliative Care Unit nurses in interviews to understand their experiences of using the delirium clinical practice guideline. [file 12904_2022_1010_MOESM4_ESM.docx]

Supplementary File 2: Delirium guideline focus group/interview guide for Palliative Care Unit (PCU) nurses.

Within our time together, we will begin with questions that focus on who you are, and your role and responsibilities on the PCU, and how delirium informs your practice. We will then move to talk about your experiences and perceptions of evidence-based guidelines, and specifically, your experiences of how the delirium clinical practice guidelines have been implemented on the PCU. It’s important for me to highlight that there are no ‘right’ answers and what is most important is for you to feel comfortable to share your thoughts and experiences openly and honestly.

1. Please share with us your role on the PCU?

Probe: Responsibilities (RN/RPN)

Length of time working on the PCU

Connection to the Interprofessional (IP) team

1. When you think about delirium and your experiences caring for someone with delirium, what elements come to mind?

Probe: Is there a particular case that comes to mind?

What were the primary issues that arose?

What things (people, processes) helped with this situation?

What things (people, processes) did not help with this situation?

What would you do differently in this situation?

1. How much of your clinical practice revolves around delirium?
2. Tell us about your overall experiences with evidence-based guidelines on the PCU?

Probe: What specific guidelines come to mind?

How do you feel overall about guidelines? (Value? Importance?)

Who generally institutes guidelines on the PCU?

How do guidelines inform (or not inform) your practice?

1. In thinking about the delirium clinical practice guideline that has been instituted on the PCU, what elements stand out the most to you?

The Delirium Practice Guideline was implemented in 5 specific blocks. We would like to talk about each of these blocks and how each of these blocks addressed your needs and concerns around managing delirium

Delirium Management Starter Kit

1. Describe the content and training that the delirium management starter kit provided?

1. As you think about your clinical practice, what elements from the delirium starter kit were particularly useful?

Probe: What really stood out for you? (and why)

How meaningful/relevant/important are these elements to your clinical practice/decision-making? (How will these inform your future practice?)

How do these elements fit into existing clinical care of delirium? (Is there any conflict or tension with other care practices?)

Was there anything missing?

Screening and Assessment – Specific to Nu-DESC

1. Describe the content and training that was provided around using the Nu-DESC?

1. As you think about your clinical practice, what elements were particularly useful?

Probe: What really stood out for you? (and why)

How meaningful/relevant/important are these elements to your clinical practice/decision-making? (How will these inform your future practice?)

How does using the Nu-DESC fit into existing clinical care of delirium?

Was there anything missing?

1. Are there any remaining areas of uncertainty or concern in relation to using the Nu-DESC in managing delirium with patients

Non-pharmacological Management – Specific to Delirium Checklist

1. Describe the content and training that was provided around using the Delirium Checklist for non-pharmacological management of delirium?
2. As you think about your clinical practice, what elements from this block were particularly useful?

Probe: What really stood out for you? (and why)

How meaningful/relevant/important are these elements to your clinical practice/decision-making? (How will these inform your future practice?)

How would using the Delirium checklist fit into existing clinical care of delirium?

Was there anything missing?

1. Are there any remaining areas of uncertainty or concern in relation to using the Delirium Checklist in managing delirium with patients, families or the IP team?

Communicating with delirious Patients

1. Describe the content and training in communicating with delirious patients
2. As you think about your clinical practice, what elements from this ‘block’ were particularly useful?

Probe: What really stood out for you? (and why)

How meaningful/relevant/important are these elements to your clinical practice/decision-making? (How will these inform your future practice?)

How would using the ‘block’ fit into existing clinical care of delirium?

Was there anything missing?

1. Are there any remaining areas of uncertainty or concern in relation to communicating with delirious patients?

Pharmacological Management – Framework to pharmacological management and information (Pharmacological Algorithm)

1. Describe the content and training that the pharmacological management block provided?

1. As you think about your clinical practice, what elements from this block were particularly useful?

Probe: What really stood out for you? (and why)

How meaningful/relevant/important are these elements to your clinical practice/decision-making? (How will these inform your future practice?)

How would using the block fit into existing clinical care of delirium?

How do they delirium CPG direct your clinical practice with regard to PRN medication?

Was there anything missing?

1. Are there any remaining areas of uncertainty or concern in relation to the pharmacological management block in managing delirium with patients, families or the IP team?

Evaluation and Monitoring –

1. Describe the content and training of the evaluation and monitoring block
2. As you think about your clinical practice, what elements from this block were particularly useful?

Probe: What really stood out for you? (and why)

How meaningful/relevant/important are these elements to your clinical practice/decision-making? (How will these inform your future practice?)

How would the evaluation and monitoring block fit into existing clinical care of delirium?

Was there anything missing?

Summing Up

1. In thinking about the overall delirium CPG, how successful do you think the implementation of the CPG has been on the PCU?
2. Thinking back to the cases you discussed at the beginning, how do you think the PCU delirium CPG would affect clinical care?
3. Any other comments surrounding the PCU delirium CPG?
