# Supplementary material for: Adaptation, implementation, and mixed methods evaluation of an interprofessional modular clinical practice guideline for delirium management on an inpatient palliative care unit
Source: BMC Palliat Care. 2022 Jul 16;21:128. doi: 10.1186/s12904-022-01010-6 (PMC9287908; doi:10.1186/s12904-022-01010-6)
Supplement: Supplementary file 5 — Additional file 5: Compiled Supplementary Tables: Additional results for the delirium clinical practice guideline evaluation survey, and pre- and post-implementation chart audit. Table 1. Respondent demographics for evaluation survey. Table 2. Patient demographics for pre- and post- delirium guideline implementation chart audit. Table 3. Chart audit results for antipsychotic and benzodiazepine medication administration 24 hours before documented delirium diagnosis. Table 4. Chart audit results for antipsychotic and benzodiazepine medication administration 0-48 hours after documented delirium diagnosis. [file 12904_2022_1010_MOESM5_ESM.docx]

# Additional file 5

Additional file 5, Table 1. Respondent demographics for evaluation survey.

|  | | **n (%)** |
| --- | --- | --- |
| **Survey response rate by respondent role** | Physicians/Pharmacist | 6/9 (67) |
|  | Nurses (RN, RPN, APN/NPL, Clinical Manager) | 18/66 (27) |
|  | Allied Health (Social worker, spiritual care provider) | 1/2 (50) |
| **Length of time working on PCU** | <5 years | 13/25 (50) |
|  | 5-10 years | 6/25 (23) |
|  | 10-15 years | 1/25 (4) |
|  | >15 years | 6/25 (23) |

Abbreviations:

APN: advanced practice nurse

NPL: nursing practice leader

PCU: palliative care unit

RN: registered nurse

RPN: registered practical nurse

Additional file 5, Table 2. Patient demographics for pre- and post-delirium guideline implementation chart audit.

|  | **Pre-delirium CPG implementation,**  **n=20 charts (%)** | **Post-delirium CPG implementation,**  **n=20 charts (%)** |
| --- | --- | --- |
| **Mean age, years (range)** | 79.0 (53-99) | 82.2 (57-95) |
| **Sex: female** | 11 (55) | 11 (55) |
| **Primary diagnosis** | | |
| Cancer | | |
| - Lung | 2 (10) | 2 (10) |
| - Breast | 1 (5) | 1 (5) |
| - Colorectal | 2 (10) | 4 (20) |
| - Pancreatic | 3 (15) | 0 (0) |
| - Gastric | 2 (10) | 1 (5) |
| - Genitourinary | 2 (10) | 4 (20) |
| - Other | 4 (20) | 4 (20) |
| Non-Cancer | | |
| - Dementia | 1 (5) | 2 (10) |
| - Frailty | 1 (5) | 1 (5) |
| - Other | 2 (10) | 1 (5) |
| **Discharge status** | | |
| Death | 17 (85) | 19 (95) |
| Discharged alive | 3 (15) | 1 (5) |
| **Mean duration of admission, days (range)** | 37 (2-215) | 18 (1-63) |
| **Delirium diagnosis method** | | |
| Documented positive CAM | 9 (45) | 11 (55) |
| Clinical diagnosis | 11 (55) | 9 (45) |

Abbreviations:

CAM: Confusion Assessment Method

CPG: clinical practice guideline

Additional file 5, Table 3. Chart audit results for antipsychotic and benzodiazepine medication administration 24 hours before documented delirium diagnosis.

|  | **Pre-CPG implementation (n=20 charts)** | | **Post-CPG implementation (n=20 charts)** | |
| --- | --- | --- | --- | --- |
|  | N (%) patients on medication | Mean dose administered over 24 hours (mg) (range) | N (%) patients on medication | Mean dose administered over 24 hours (mg) (range) |
| **Scheduled (regular) medications** |  |  |  |  |
| - Haloperidol | 3 (15) | 1.13 (0.50-2.00) | 1 (5) | 0.50 |
| - Methotrimeprazine† | 3 (15) | 6.50 (2.00-12.5) | 1 (5) | 2.50 |
| - Quetiapine | 0 (0) | 0.00 | 1 (5) | 25.0 |
| - Midazolam‡ | 0 (0) | 0.00 | 0 (0) | 0.00 |
|  |  |  |  |  |
| **PRN/ Stat medications** |  |  |  |  |
| - Haloperidol | 3 (15) | 0.50 (0.00-1.00) | 6 (30) | 0.11 (0.00-0.50) |
| - Methotrimeprazine† | 4 (20) | 3.31 (0.00-6.25) | 1 (5) | 0.00 |
| - Quetiapine | 0 (0) | 0.00 | 0 (0) | 0.00 |
| - Midazolam | 1 (5) | 0.50 | 2 (10) | 1.50 (0.00-3.00) |
|  |  |  |  |  |
| **No medications** | 9 (45) |  | 13 (65) |  |

† Called levomepromazine in some countries

‡ On our unit, scheduled midazolam is administered as a continuous infusion using a continuous ambulatory delivery device.

Note: For purposes of calculating mean dose of haloperidol and methotrimeprazine, the doses by oral and subcutaneous routes were taken as equivalent, e.g. haloperidol 0.5mg PO ≡ 0.5mg subcut. (Haloperidol is usually ordered as 0.5mg PO or subcut on our unit).

Abbreviations: CPG: clinical practice guideline; PO: by mouth; PRN: pro re nata (‘as needed’); subcut: subcutaneous

Additional file 5, Table 4. Chart audit results for antipsychotic and benzodiazepine medication administration 0-48 hours after documented delirium diagnosis.

|  | **Pre-CPG implementation (n=20 charts)** | | **Post-CPG implementation (n=20 charts)** | |
| --- | --- | --- | --- | --- |
|  | N (%) patients on medication | Mean dose administered over 48 hours (mg) (range) | N (%) patients on medication | Mean dose administered over 48 hours (mg) (range) |
| **Scheduled (regular) medications** |  |  |  |  |
| - Haloperidol | 10 (50) | 2.81 (0.50-8.00) | 6 (30) | 1.75 (1.00-3.00) |
| - Methotrimeprazine† | 10 (50) | 20.09 (5.00-112.5) | 6 (30) | 19.06 (2.5-60) |
| - Quetiapine | 2 (10) | 43.75 (12.5-75.0) | 0 (0) | 0.00 |
| - Risperidone | 0 (0) | 0.00 | 1 (5) | 0.50 |
| - Midazolam‡ | 0 (0) | 0.00 | 1 (5) | 6.63 |
| - Trazodone | 0 (0) | 0.00 | 1 (5) | 75.00 |
|  |  |  |  |  |
| **PRN/ Stat medications** |  |  |  |  |
| - Haloperidol | 12 (60) | 3.23 (0.00-8.00) | 13 (65) | 0.81 (0.00-2.00) |
| - Methotrimeprazine† | 9 (45) | 14.73 (0.00-37.5) | 6 (30) | 5.00 (0.00-15.0) |
| - Quetiapine | 0 (0) | 0.00 | 0 (0) | 0.00 |
| - Midazolam | 6 (30) | 3.94 (0.00-10.0) | 12 (60) | 1.96 (0.00-11.0) |
| - Trazodone | 0 (0) | 0.00 | 1 (5) | 12.50 |
|  |  |  |  |  |
| **No medications** | 1 (5) |  | 1 (5) |  |

†Called levomepromazine in some countries

‡ On our unit, scheduled midazolam is administered as a continuous infusion using a continuous ambulatory delivery device.

Note: For purposes of calculating mean dose of haloperidol and methotrimeprazine, the doses by oral and subcutaneous routes were taken as equivalent, e.g. haloperidol 0.5mg PO ≡ 0.5mg subcut. (Haloperidol is usually ordered as 0.5mg PO or subcut on our unit).

Abbreviations: CPG: clinical practice guideline; PO: by mouth; PRN: pro re nata (‘as needed’); subcut: subcutaneous
